# Giving Voice to Clinical Study Participants: Development and Deployment of Sequential Patient Experience Surveys for Global Clinical Studies

**DOI:** 10.1007/s43441-020-00115-5

**Published:** 2020-01-21

**Authors:** Elizabeth Manning, Mitch Herndon, Wendy Frye, Tammy S. Ice, Nadia Thyssen, Daphnee S. Pushparajah, Stephen L. Yates

**Affiliations:** 1 UCB Pharma, 8010 Arco Corporate Drive, Raleigh, NC 27617 USA; 2 Accelerated Enrollment Solutions, Pharmaceutical Product Development, LLC, Wake Forest, NC USA; 3 UCB Pharma, Brussels, Belgium; 4 Alexion Pharmaceuticals, Hayes, UK

**Keywords:** Patient-centric, Patient participation, Patient engagement, Social sciences, Attitudes surveys, Questionnaires

## Abstract

**Background:**

Biopharmaceutical companies are piloting patient experience surveys (PES) to help enhance patient satisfaction with clinical studies. However, most PES have been conducted at study close-out, which can hinder recall and responsiveness, and at a limited number of sites, which restricts their applicability to global studies. Our aim was to investigate the feasibility of developing sequential PES, which would be deployed globally, and to provide practical recommendations based on our real-world experience.

**Methods:**

To develop sequential PES (introductory, interim, close-out), we customized a previously developed patient experience close-out survey. Extensive input was gained from multiple stakeholders (e.g., survey experts, patient advisors, psychometricians, clinical trialists, lawyers). To deploy the PES in global studies, we prepared PES-specific ethics committee submissions, training materials (e.g., slides, videos), and PES invitation aids (postcards, digital app reminders).

**Results:**

Developing and deploying sequential PES in global clinical studies was feasible. The 3-part online PES (25 to 37 questions per survey) passed health literacy testing. To facilitate benchmarking, the PES included core questions (including a Net Promoter Score question). The PES gained ethics approval and was deployed globally in 2017–2018 in 12 phase 2 and 3 clinical studies in North America, Europe, and the Asia–Pacific. Based on the real-world insights gained and the challenges encountered, we have made recommendations for PES.

**Conclusions:**

Our practical recommendations on the development and deployment of sequential global PES may assist others to implement PES efficiently and effectively, allowing them to gain feedback from patients globally during clinical studies.

**Electronic supplementary material:**

The online version of this article (10.1007/s43441-020-00115-5) contains supplementary material, which is available to authorized users.

## Introduction

Over the past 20 years, the increasing complexity of clinical studies has slowed the development of medicines [1, 2]. Complex clinical studies place a strain on all involved, including patient participants [1, 3, 4]. Not surprisingly, poor clinical study experiences can hinder recruitment and retention, which can then lead to study delays and increased costs [5–8].

There is increasing recognition that structured and systematic patient engagement may help improve clinical studies [9–13]. Initiatives from the Food and Drug Administration (Patient Focused Drug Development) [11], Clinical Trials Transformation Initiative [9, 10], European Patients’ Academy on Therapeutic Innovation (EUPATI) [14], TransCelerate Biopharma Inc. (Patient Experience Initiative) [15], Innovative Medicines Initiative (Patients Active in Research and Dialogues for an Improved Generation of Medicines—PARADIGM) [12], and Patient Focused Medicines Development [16], among others, aim to build practical frameworks to guide patient engagement.

One practical way to engage patients in clinical studies is to ask for their feedback. Indeed, industry sponsors are now being encouraged to measure the extent of patient engagement in studies, with patient feedback identified as a suitable metric [17]. Feedback can be gained through patient experience surveys (PES), and early research has shown that PES can be implemented at the start or end of a study [18], or at both time points in a single-center setting [19]. However, further real-world research is needed on PES [11], particularly, the use of sequential surveys within a study and within a global setting. Sequential surveys offer several advantages, including shorter patient recall time, the potential to correct poor patient experiences in near real time, and the measurement of patient satisfaction relative to their expectations. In terms of real-world relevance, as most sponsors conduct global studies, insights from PES research that take operational and cultural issues into account are needed.

As part of its overarching patient partnering strategy for medicines development [11, 13, 20], UCB Pharma is now implementing the structured and systematic use of PES. Successful development and deployment of PES in selected studies could give a voice to and benefit many participants around the world. Given the call for industry sponsors to share details on the operational approaches used to engage patients [17], UCB Pharma is committed to sharing real-world insights gained from our PES research. Doing so could help other sponsors introduce PES and contribute to an industry-wide effort to improve clinical studies by listening to and acting on patient feedback.

The aim of the current project was to investigate the feasibility of developing and deploying PES for use at 3 time points within global clinical studies and to share real-world recommendations to guide the future use of PES. This paper describes the processes and offers practical recommendations for developing and deploying PES; we intend to discuss outcomes from the surveys themselves in a subsequent paper.

## Methods

### Design and Oversight

**Figure 1.**
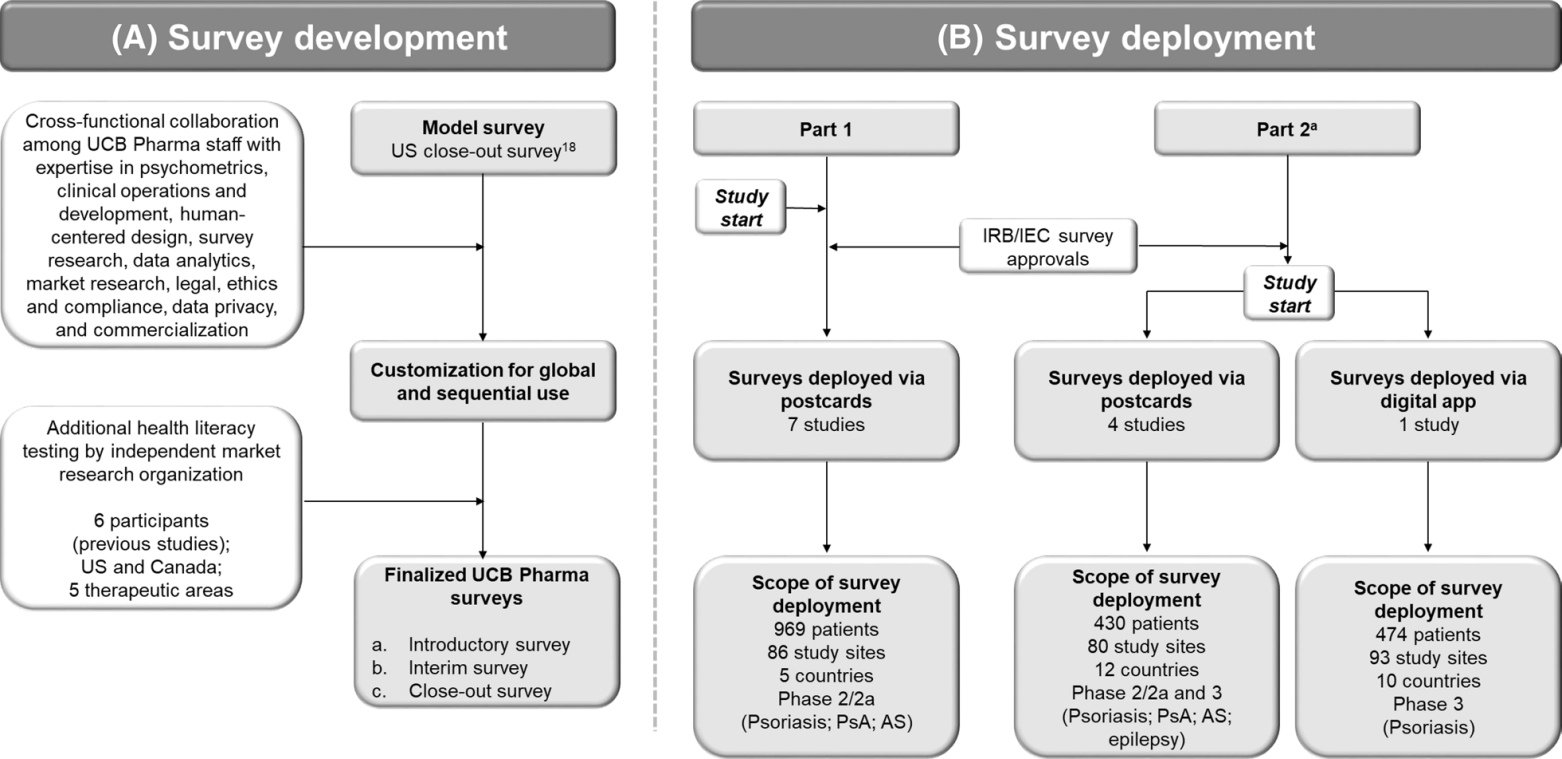
Schematic of the Survey Development and Deployment Stages. Note that some study sites may be involved in more than one study. ^a^Ongoing. *AS* ankylosing spondylitis, *IEC* independent ethics committee, *IRB* institutional review board, *PsA* psoriatic arthritis, *US* United States.

This project comprised both a survey development stage, in which the questions were created, and a subsequent two-part deployment stage, in which surveys were used in clinical studies (Fig. 1). The project was organized and overseen by a cross-functional taskforce within UCB Pharma using a disease-agnostic approach and conducted in accordance with ethical standards for market research and data privacy regulations. Alignment with UCB Pharma best practice for protocol simplification meant that the PES (which were not associated with study endpoints) were not included as a required activity within the clinical study schedule of events. Thus, potential responders had to provide separate consent before participation. When the online surveys were made available to patients, the first page (landing page) explained the survey aim, UCB Pharma sponsorship, and assurances around confidentiality and informed consent. All participants who viewed the landing page at the end of their study participation were offered the opportunity to opt-in to undergo an interview and receive plain language summaries, even if they chose not to complete the survey. No monetary incentives were provided for survey participation.

### Survey Development

In 2016, UCB Pharma started development of PES, which were modeled on a self-administered close-out survey designed by an independent market research organization [18]. That model survey was informed by a literature review and patient interviews, and included a 50-item set of closed-ended questions (multiple choice or 5-point Likert scale) on the themes (identified as preferred from patient interviews) of communication, site experience, convenience, relationship building and support, compensation, and helping self and others [18].

Recognizing the importance of capturing the patient experience at key moments during their clinical study journey, UCB Pharma planned to develop 3 separate surveys: (1) “Introductory survey,” administered after informed consent, (2) “Interim survey,” administered during the clinical study at a time determined by the study team, and (3) “Close-out survey,” administered at the end of study participation (i.e., at study completion or patient withdrawal). For each survey, UCB Pharma adapted the model survey by adding and reformulating questions to fulfill UCB Pharma’s strategy to partner with patients. An iterative process was used involving an internal, cross-functional team with expertise in psychometrics, global clinical operations and development, human-centered design, survey research, data analytics, market research, legal, ethics and compliance, data privacy, and commercialization management.

The new survey questions underwent health literacy testing via a patient engagement co-creation process conducted by the independent market research organization on UCB Pharma’s behalf. An online survey/telephone interview was administered to a sample of 6 past clinical study participants (4 women, 2 men, all Caucasian, aged 36–64 years) residing in the United States (US) and Canada who were recruited through a proprietary database. The new questions were then critically evaluated based on comprehensive review of patient feedback.

### Survey Deployment

#### Part 1

Between March and August of 2017, UCB Pharma deployed the PES in 7 global phase 2/2a studies at sites in the US, Canada, Poland, Australia, and Japan (Fig. 1). The planned use of the PES was included in a submission to independent ethics committees (IECs) after the study had started. The PES were submitted to a central institutional review board (IRB) in the US first. To establish precedent and enable a request for no changes to the surveys, the US IRB approval was included in subsequent IRB/IEC submissions. Ethics committee approvals were granted before the PES were initiated. Training materials on the PES, including their purpose and deployment, were distributed to sites via email and/or video. Study participants were invited to participate in the online survey via a printed postcard (in the participant’s own language), which guided the participant to the online PES landing page. Site staff were asked to distribute postcards to all study participants at the appropriate study visit. Because the PES were not included in the study protocol, site staff were also sent reminders (e.g., direct-to-site letters, study portals, newsletters) about survey distribution. The UCB Pharma Site Engagement Team was asked to obtain feedback from site staff on survey deployment.

#### Part 2

Following the initial deployment in Part 1, the PES were deployed at the start of 5 phase 2/2a and 3 global clinical studies between January and August 2018 (Fig. 1). All 5 studies in Part 2, as well as 2 of 7 studies in Part 1, are ongoing as of September 2019. The process of survey deployment in Part 2 was modified slightly based on experience obtained during Part 1. Site staff training materials were similar to those in Part 1; however, unlike in Part 1, the PES were included in the initial (i.e., before study start) IRB/IEC submissions, discussed during site initiation visits and prestudy investigator meetings, and deployed at the start of each study.

Most studies in Part 2 used postcard invitations, as in Part 1. However, in one study, a “myUCB 4me” digital app was developed by UCB Pharma to support clinical outcome assessments. The app was optimized to issue the PES invitations at the appropriate study visit and direct participants to the landing page so that they could access the survey on the same device. Site staff and UCB Pharma staff were interviewed to gain feedback on PES deployment.

The surveys were translated by qualified translation services into 6 languages or local dialects (e.g., Canadian French, Australian English) for Part 1 and 18 languages/dialects for Part 2. The meaning of the questions was checked when translated into other languages, including via back-translation.

### Data Management and Statistics

A data management plan was developed before the study that addressed the confidentiality, acquisition, management, analysis, storage, reporting, and sharing of survey data. An invitation-only system was used to prevent unauthorized PES access; study participants could only participate if they received a postcard invitation from site staff or an invitation from the myUCB 4me app.

To reassure participants regarding confidentiality, no identifying information was collected. Therefore, individual participant responses could not be linked from one survey to the next survey. Site-specific feedback was only available once at least 5 surveys had been completed and was provided in aggregate form to protect patients from being identified. This threshold also applied at the study level and country level.

## Results

### Survey Development

Development of sequential PES for global studies was feasible, and insights gained from this real-world experience enabled us to develop practical recommendations for others who may wish to develop PES (Table 1).

**Table 1.** Recommendations for the Development of Sequential Patient Experience Surveys (PES) in Global Clinical Studies.

1. Identify a PES champion who can drive innovation and a patient-centric culture positively, within your organization, with your service providers, and with clinical study site staff
2. Establish a clear and concise rationale for the “why” and the “how” of the surveys (e.g., purpose, type, timing, and number of surveys). Ensure this rationale is clearly and proactively communicated to all stakeholders (e.g., IRB/IECs, site staff, patients and advocates, internal teams) to help reduce concerns and queries. Communication should be tailored to each stakeholder’s priorities and perspective
3. Co-develop a plan with stakeholders who are directly involved in deployment to manage the risks of PES (e.g., survey fatigue, operational challenges, cultural considerations, and the potential impact on clinical study data)
4. Plan and document the timelines and resources required to develop surveys that will enable efficient and effective alignment with clinical study timelines (e.g., surveys ready for submission to ethics committees, globally)
5. Develop robust and relevant surveys by working with PES consultants, survey and market research experts, and key stakeholders, including representative patients (and caregivers, where relevant) and clinical study professionals (sponsors and site staff, legal and compliance advisors)
6. Prioritize ‘core questions’ that will enable internal and external benchmarking (e.g., across time, sponsors, sites, therapeutic areas). Core questions for PES are emerging through multi-sponsor initiatives
7. Limit ‘sponsor-specific questions’ to those that address unique and important issues for your organization
8. Limit ‘study-specific questions’ to maintain efficiencies related to PES standardization (e.g., translations, document preparation)
9. Identify countries that will most likely use the survey and ensure valid translations of the survey can be developed. Central funding of translations for countries commonly involved in studies can reduce burden at the study level
10. Ensure that questions intended for pediatric studies are tested for health literacy in a relevant population (e.g., adolescents)
11. Identify survey metrics that should be reported (according to best practice reporting of surveys) and ensure the survey tool used can provide the necessary data, reliably and efficiently (e.g., via a visual dashboard)
12. Put processes in place to capture and share, internally and externally, insights gained from the survey development process to enhance future surveys

*PES* Patient Experience Surveys, *IEC* independent ethics committee, *IRB* institutional review board.

All surveys retained core questions from the model close-out survey [18] to enable future benchmarking, but questions were tailored and built upon to suit the timing and intentions of each survey (Table 2). Thus, the Introductory survey was designed to gather information on patient expectations and initial experiences of the study (e.g., the informed consent process). The Interim survey was designed to evaluate if expectations were being met and if communication and interactions with site staff were satisfactory and appropriate. The Close-out survey reassessed some of the themes raised in the Introductory and Interim surveys to capture overall patient perspectives and offered participants opportunities to receive follow-up information.

**Table 2.** Themes Addressed in the 3 Patient Experience Surveys and Example Questions.

Theme	Introductory Survey	Interim Survey	Close-Out Survey	Example Questions
Patient profile	•	•	•	How far do you live from the research study site?
Awareness and participation	•	•		How did you hear about the research study?
Overall experience	•	•	•	To what extent has the research study experience met your expectations?
Recruitment process	•			When you think about how you first heard about the research study, how satisfied were you with the clarity of information provided?
Informed consent	•			Was the time spent explaining the informed consent process too long, too short, or just right?
Communication preferences	•			What might be helpful? Options provided, e.g., brochure, website, telephone helpline
Involvement and interpersonal engagement		•	•	How satisfied were you with the level of dignity and respect with which you were treated?
Site instructions		•		How satisfied were you with the level of preparedness of the research study staff with each of your visits?
Medications usage		•		How satisfied were you with the ease of opening your study medication?
Information and communication			•	How satisfied were you with the information you received about the different research study procedures?
Information follow-up after leaving the study			•	If it were to be available, would you like to receive any of the following information?
Infrastructure, logistics, and comfort			•	How satisfied were you with the overall length of an average study visit?
Communication after leaving the study			•	If it were to be available, would you like to receive any of the following information? Options provided, e.g., easy to understand study results, opportunities to participate in future studies
Consent to receive follow-up opportunities			•	I give permission to receive follow-up information that may become available related to my research study participation, such as the research study status or research study results
Total no. of questions^a^	25	27	37	

^a^Not all questions will be answered by every participant (questions may be automatically skipped depending on previous answers).

For all 3 surveys, UCB Pharma included a Net Promoter Score (NPS) question: “Based on your experience in this research study, how likely is it that you would recommend participating in a research study to another person?” This question offers the opportunity to gauge patient satisfaction with clinical study participation using a measure that has been validated in customer-focused industries [21].

The final surveys consisted of 25 (Introductory survey) to 37 (Close-out survey) questions that were expected to take 5–10 min to complete (Table 2). Response options were fixed and included multiple choice, a 5-point Likert scale (ranging from “not at all satisfied” to “completely satisfied”), and “not applicable” elements. Within each survey, question order was fixed; however, for multiple-choice questions, choice order was randomized to minimize bias. Multiple answers were permitted for some questions.

Because each survey (Introductory, Interim, Close-out) was completed independently and sequential surveys from individual participants were not linked, we included an initial question asking participants how long they had been in the study. The participant was then directed to the relevant survey based on whether they had just started the study, had been in the study for some time, or had completed the study (i.e., an adaptive questioning process was used). Each survey included a completion bar visible to the participant, and all questions needed to be answered before completion. Participants were able to review and change their answers before survey completion.

Adaptations implemented between Part 1 and Part 2 included the option for a caregiver to answer on behalf of the study participant and the creation of a separate caregiver experience surveys; both adaptations were to support 2 pediatric studies in Japan. There was one minor change in the PES itself during transition from Part 1 to 2: we deleted the phrase “sugar pill” after the word *placebo* in an answer to one question, which also allows the PES to be used for clinical studies of medications other than pills (e.g., injections).

Gaining support for the development of the PES from internal teams (e.g., regulatory, legal, clinical), and especially the study teams, was critical. The PES involved a change in a highly regulated and deeply embedded clinical trial process, but alignment with UCB Pharma’s commitment to patient engagement and patient experience facilitated acceptance.

### Survey Deployment

Deployment of sequential PES in global clinical studies was possible, and we refined the process between Parts 1 and 2. We have made practical recommendations for PES deployment, which may assist others considering how to implement PES (Table 3).

**Table 3.** Recommendations for the Deployment of Sequential Patient Experience Surveys (PES) in Global Clinical Studies.

1. Identify survey deployment champions at each site and within CRO teams; they should understand the importance of PES and be able to drive efficient and effective deployment
2. Ensure study teams, site staff, and CRO teams are aware of and proactively budget for PES requirements
3. Leverage technology (e.g., digital app for the clinical study) to enhance the deployment process, reduce burden for site staff, and facilitate survey participation
4. Prepare training materials, operational aids, reminders, and motivational communications to help site staff understand the purpose of the PES and remember the process. Response rates and recall information may be enhanced if site staff encourage study participants to complete the survey after the relevant visit
5. Translate and adapt content, as well as the deployment process, to take into account cultural preferences (e.g., Japanese sites may prefer more detailed documentation than US sites)
6. Include a concise, practical-focused review of the survey deployment process in the clinical study investigator meeting and the study start-up visit at each site
7. Proactively seek out feedback from site staff, CRO teams, and patient advisors and consider if/how to adjust the deployment process before or soon after patient recruitment starts
8. Ensure each site has all the necessary approvals in place and training materials required so that surveys can be deployed at the start of recruitment. Consolidating all PES material (e.g., sequential surveys, patient communication materials) in one document for IRB/IEC review can enhance efficiency
9. Put processes in place to capture, share, and respond to insights gained from the survey deployment process (e.g., increase process consistency to enhance scalability) in future studies

*CRO* contract research organization, *PES* Patient Experience Surveys, *IEC* independent ethics committee, *IRB* institutional review board.

#### Part 1

Part 1 survey deployment was generally well received by the IRBs/IECs, even though the PES concept was new. A Canadian IRB requested more clarity regarding the data sharing described on the landing page. For transparency, UCB Pharma revised the landing page before pursuing other IRB/IEC submissions. This process, in addition to some studies operating within accelerated timelines, meant that the PES were not available for use at the same study time point across all sites.

Deployment of surveys in a clinical study already underway was operationally challenging. For example, even with experienced site staff, adding another “procedure” (the PES) when they were already focused on protocol-related procedures was not ideal.

#### Part 2

During Part 2, we made several operational changes to overcome challenges identified in Part 1. First, we included the PES in the initial IRB/IEC submissions and presented the PES to sites as part of the initiation process for the clinical study. Second, we also provided the sites and contract research organizations with more information on the value of the PES, as well as additional training to strengthen their engagement. Third, development of the digital myUCB 4me interface was introduced to improve the ease of completing the PES and to enhance tracking of deployment and metrics.

The PES were approved by most IRBs/IECs, but were not approved in Belgium where some IECs requested that the surveys be included in the informed consent forms. To maintain consistency across countries and study sites, the informed consent form was not changed to meet this specific request. Some Japanese IECs also rejected the submissions, partly because the PES were not included in the protocol, but also because of concerns with the use of patients’ private devices for the PES. Site staff suggested that the use of sponsor devices or paper-based surveys would be viewed more favorably, as would inclusion of the PES in the study protocol.

Although Part 2 is ongoing, we anticipate that the myUCB 4me app will enable efficient calculation of response rates and early experience suggests that the app may enhance uptake.

## Discussion

To our knowledge, this is the first demonstration of the feasibility of developing and deploying sequential PES for use in a global setting. Based on our real-world experience, we have made practical recommendations to assist others (Tables 1 and 3). Our initial observations may help prompt and guide further PES research so that the clinical research community can build a robust evidence base to support PES.

Gaining input from patients is critical for improving the focus and conduct of clinical studies, and patient involvement in the development of PES represents one such step. Former study participants contributed to the development of both the model close-out survey and UCB Pharma’s customized sequential PES. Given the value of patient feedback, we are committed to ensuring future PES have patient input, particularly early in the process and from a diverse range of patients.

Consistent with recent recommendations on metrics and the importance of gaining direct feedback from patients, we introduced the NPS in our PES. The NPS is a single quantitative measure that can potentially serve as a valuable benchmarking metric. Although the NPS has been widely adopted in many customer-focused industry sectors [21], its use in clinical studies is relatively recent [22]. Further research, with patient partners, on the value and validity of the NPS in PES is needed, but as a simple and straightforward metric, the NPS may appeal to clinical study participants, site staff, and sponsors.

Given the global reach of many clinical studies, we sought to develop and deploy PES suitable for global use. This type of PES offers the potential to gain feedback from larger and more representative samples of study participants [11]. Using a structured and systematic approach facilitates the global deployment of PES, but unexpected challenges can occur. As global deployment is an innovative step in PES, it is inevitable that organizational change management issues will arise—for site staff, IRB/IECs, and sponsors. Our patient engagement team worked with internal and external stakeholders to streamline the international implementation of this innovation. Efficient review, approval, and implementation of global PES could be enhanced by proactive, supportive, and goal-focused change management practices by each stakeholder. Deployment of global PES also means that country-specific needs and preferences must be accommodated. For example, in Japan, study sites required more detailed, country-specific, written documentation that considered their cultural norms. Although PES have been conducted at a national level in Japan [23], there is limited research on PES conducted by industry sponsors in this country. Further, patient feedback to healthcare professionals is not a common practice in Japan [24]. Care is also needed regarding the language and terminology used when communicating with patients via a survey across multiple geographies. For example, the landing page of each survey informed patients that their responses would be treated anonymously. However, in Canada, the word *anonymous* has a strict legal definition that was not appropriate in the PES and was changed to *confidential*.

The multi-sponsor use of PES is likely to be enhanced by the forthcoming release of an industry framework and guidance called the Study Participant Feedback Questionnaire toolkit [25]. This toolkit is being developed by members of TransCelerate Biopharma Inc., an international not-for-profit organization facilitating collaboration among industry sponsors. Broad uptake of a standard survey framework could facilitate industry-wide benchmarking of the patient experience in clinical studies. This timely support for PES in clinical studies coincides with the increasing recognition of the value of patient engagement across the entire medicines’ development lifecycle [12, 26].

Our recommendations for PES need to be considered in the context of a number of limitations. Although the site staff and service providers we partnered with have worked with many sponsors, our recommendations do reflect the experience of just one sponsor, UCB Pharma. As other sponsors introduce PES in clinical studies, we expect and encourage updates to our original set of recommendations. Further, as our PES were limited to certain therapeutic areas and clinical study phases, adaptations would need to be considered for other studies (e.g., acute conditions, phase 1 studies). We also recognize that our global PES experience was not entirely global. Although we deployed the PES in North American, European, and Asia–Pacific regions, the need to take country-specific practices into account may affect the applicability of some of our recommendations. We also acknowledge that insights from further research may affect our recommendations. Like regulators and other stakeholders who are using innovative ways to listen to and act on patient feedback, we appreciate that the “science of patient input is constantly evolving” [11]. As with any innovation, research will be required to address the questions that will arise as implementation experiences are shared.

## Conclusions

As part of its commitment to patient engagement in medicines development, UCB Pharma has developed and deployed sequential PES across several global clinical studies. Deploying PES at multiple time points during a clinical study may help sponsors better understand how to improve the study processes that are most important to patients at each stage. Moreover, global PES deployment is possible, provided that operational challenges and cultural differences are addressed appropriately and proactively whenever possible. Based on this real-world experience, we have provided recommendations that may help others to implement PES. The veracity and value of these recommendations may be tested or enhanced as the UCB Pharma PES are further refined and as other sponsors deploy PES.

## Electronic supplementary material

Below is the link to the electronic supplementary material.

Supplementary material 1 (PDF 1840 kb)
