# Supplementary material for: Giving Voice to Clinical Study Participants: Development and Deployment of Sequential Patient Experience Surveys for Global Clinical Studies
Source: Ther Innov Regul Sci. 2020 Jan 21;54(5):1001–9. doi: 10.1007/s43441-020-00115-5 (PMC7458896; doi:10.1007/s43441-020-00115-5)
Supplement: Supplementary file 1 — Supplementary material 1 (PDF 1840 kb) [file 43441_2020_115_MOESM1_ESM.pdf]

This summary has been prepared using the Plain Language Summaries Toolkit,
co-created with patients http://www.envisionthepatient.com/plstoolkit

How to prepare and use Patient Experience Surveys in global clinical studies

The purpose of this plain language summary is to help you understand recent research about Patient Experience Surveys.

Key Points

- A Patient Experience Survey helps people tell researchers what it was like to take part in a clinical study.
- The research project reported here:
  - Showed that researchers can prepare a Patient Experience Survey that can be used at the start, during, and at the end of global clinical studies.
  - Identified ways that might help other researchers to prepare and use Patient Experience Surveys.
- Research on Patient Experience Surveys is still new. Other research projects on surveys might give different results.

1 What is a Patient Experience Survey?

- A Patient Experience Survey is like a customer satisfaction survey.
- It helps people tell researchers what it was like to take part in a clinical study.

2 Why are Patient Experience Surveys needed?

Researchers do clinical studies to help develop new medicines.

But, over time, these studies have become:

LONGER | HARDER | MORE COSTLY

Feedback from people who take part in studies might help researchers improve how they do these studies.

> **"I would actually be pretty happy to make the study better. Our outside perspective should help improve putting on a project like this."**
>
> *Feedback from a patient helping to prepare a Patient Experience Survey*

3 What did this research project look at?

- So far, most research on Patient Experience Surveys has looked only at surveys done:
  - At the end of a study.
  - At a few clinical study sites.
- In this research project, researchers prepared and used a Patient Experience Survey:
  - At the start, during, and at the end of a study.
  - In clinical studies at many sites across the world.

4 Who took part in this research project?

Preparing the survey

- People who had taken part in clinical studies, clinical study experts, and survey experts helped prepare the survey.

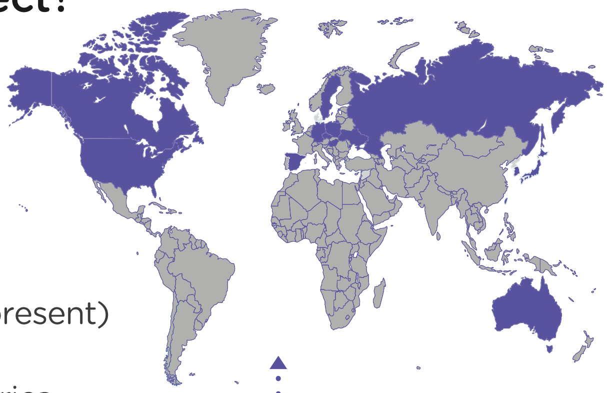

Using the survey

- People taking part in 12 clinical studies (2017-present) used the surveys.
- These clinical studies were done in North America, Europe, and the Asia Pacific Region.
- Survey participation continues with several ongoing clinical studies.

5 What were the results of this research project?

Researchers found it was possible to prepare and use Patient Experience Surveys

- at the start, during, and at the end of a clinical study
- in global clinical studies

Researchers made the following suggestions to help other researchers do surveys:

To prepare a survey

people

- Identify a survey champion who can motivate study teams* and explain the 'why' and 'how' of the survey.
- Involve patients and study teams who will use the survey.
- Involve patients and study teams from countries that will take part in the global studies.

time & money

- Fund experts to help you prepare and test the survey.
- Fund translation of the survey into other languages.
- Send the survey on time with other study documents to ethics committees.†
- Fund a tool to help you check the success of your survey.

other

- Include questions that will allow you to compare results with other surveys.
- Have a plan to manage risks (eg, forgetting to do the survey).
- Share what you learned from preparing the survey.

To use a survey

people

- Identify a survey champion who can help study teams understand the importance of the survey and how to use it.
- Make sure study teams know how to include a survey in their budgets.
- Early in the study, ask study teams for feedback on using the survey.

time & money

- Fund a tool to help patients use the survey, quickly and easily.
- Prepare training materials about the survey for study teams and patients.
- Fund translation of training materials into other languages.

other

- Share what you learned about using the survey.
- Protect patient privacy and ensure feedback is confidential.

**Study teams: Doctors and nurses at clinical study sites who do the study and service providers who check how well the study is going.*
†Ethics committee: Helps protect the rights and safety of people taking part in a clinical study.

6 Are there plans for further research projects?

Yes, UCB Pharma plans to share more learnings about Patient Experience Surveys in the future

7 Who funded this research project?

UCB Pharma, Belgium

For more information about this research project, please contact:

Elizabeth Manning
elizabeth.manning@ucb.com
+1 (919) 767 2522

Further Information

For more information about medicines development, please visit:

The European Patients Academy **https://www.eupati.eu/**

The US Food and Drug Administration **https://www.fda.gov/forpatients**

The full title of this article is: Giving voice to clinical study participants: Development and deployment of sequential patient experience surveys for global clinical studies.
You can access the full article for **free** here: **https://doi.org/10.1007/s43441-020-00115-5**

This summary was prepared by Professor Karen Woolley, PhD, CMPP from Envision Pharma Group and was reviewed by patients and plain language specialists. The summary was funded by UCB Pharma. The original authors of the full article were involved in preparing this summary.
